# Exosomal circLPAR1 Promoted Osteogenic Differentiation of Homotypic Dental Pulp Stem Cells by Competitively Binding to hsa-miR-31

**DOI:** 10.1155/2020/6319395

**Published:** 2020-09-28

**Authors:** Liangkun Xie, Zheng Guan, Mingzhu Zhang, Sha Lyu, Nattawut Thuaksuban, Suttatip Kamolmattayakul, Thongchai Nuntanaranont

**Affiliations:** ^1^Department of Oral Implantology, The Affiliated Stomatology Hospital of Kunming Medical University, Kunming, China; ^2^Department of Oral and Maxillofacial Surgery, Faculty of Dentistry, Prince of Songkla University, Hatyai, Songkhla, Thailand; ^3^Biomedical Research Center, The Affiliated Calmette Hospital of Kunming Medical University (the First Hospital of Kunming), Kunming, China; ^4^Department of Periodontology, The Affiliated Stomatology Hospital of Kunming Medical University, Kunming, China; ^5^Faculty of Dentistry, Bangkok Thonburi University, Bangkok, Thailand

## Abstract

Human dental pulp stem cells (DPSCs) hold great promise in bone regeneration. However, the exact mechanism of osteogenic differentiation of DPSCs remains unknown, especially the role of exosomes played in. The DPSCs were cultured and received osteogenic induction; then, exosomes from osteogenic-induced DPSCs (OI-DPSC-Ex) at different time intervals were isolated and sequenced for circular RNA (circRNA) expression profiles. Gradually, increased circular lysophosphatidic acid receptor 1 (circLPAR1) expression was found in the OI-DPSC-Ex coincidentally with the degree of osteogenic differentiation. Meanwhile, results from osteogenic differentiation examinations showed that the OI-DPSC-Ex had osteogenic effect on the recipient homotypic DPSCs. To investigate the mechanism of exosomal circLPAR1 on osteogenic differentiation, we verified that circLPAR1 could competently bind to hsa-miR-31, by eliminating the inhibitory effect of hsa-miR-31 on osteogenesis, therefore promoting osteogenic differentiation of the recipient homotypic DPSCs. Our study showed that exosomal circRNA played an important role in osteogenic differentiation of DPSCs and provided a novel way of utilization of exosomes for the treatment of bone deficiencies.

## 1. Introduction

Dental pulp stem cells (DPSCs) can differentiate into odontoblasts, chondrocytes, adipocytes, and osteoblasts [1, 2]. Besides those properties, DPSCs have unique characters compared with other sources of mesenchymal stem cells (MSCs), being more accessible with minimal trauma and having shown great potentiality in regenerative medicine for the treatment of various human diseases such as bone deficiencies [3]. The primary goal to regenerate new bone is to activate osteogenic differentiation of certain somatic stem cells such as bone marrow mesenchymal stem cells (BMSCs) [4]. Unfortunately, the exact mechanism of osteogenic differentiation is unclear, which might be one significant hurdle has to be overcome in order to achieve optimal clinical outcomes of bone augmentation. While DPSC as a high-potential candidate for bone regeneration, the mechanism for osteogenic differentiation of DPSC has to be further studied for better utilization of DPSC in bone regenerative medicine.

Discovered more than three decades ago, exosomes were initially regarded as a waste product releasing way by tiny vesicles composed of plasma membrane [5]. Recent years, dramatically increased exosome studies shed the light on profound functions of exosomes. Different types of RNAs in exosomes are key factors contributed to those functions of exosomes, such as exosomal microRNA (miRNA) which showed important roles in osteogenic differentiation [6]. Early in 2014, variations of expression of exosomal miRNAs derived from human BMSCs during osteogenic differentiation were found [7]. Later, the change of miRNA expression in exosomes from mineralizing osteoblasts was also found and the exosomal miRNAs showed osteogenic promotion effects [8]. Recently, MSC-derived exosomal miRNA let-7 was found to have positive role in osteogenesis [9]. However, miRNA is not the only RNA inside the exosome [10], and the roles of other types of exosomal RNAs on osteogenic differentiation need further studied.

Circular RNA (circRNA) is a serendipitous discovery in a study of human tumor suppressor gene (DCC) initially investigating exon connectivity by reverse transcription polymerase chain reaction (RT-PCR) [11]. However, the importance of circRNA had not gained attention in biological field until a number of studies showed that circRNA widely existed in different cells of human [12] and was specifically expressed in certain types of cell [13] and notably stable [12]. Recently, similar to the changed exosomal miRNA expression during certain cells' osteogenic differentiation, circRNA expression also showed a change during osteogenic differentiation [14]. Circular RNA CDR1 had an miR-7 sponge effect that positively facilitated osteogenic differentiation of periodontal ligament stem cells [15]. Nevertheless, circRNA linked to miRNA and message RNA (mRNA) as an axis [16]. However, due to intricate expression of circRNAs in osteogenic differentiation, the role of circRNAs in osteogenic differentiation has to be further investigated.

In this study, we studied the altered expression of exosomal circRNA derived from DPSCs under osteogenic induction, further demonstrated a circRNA affected osteogenic differentiation of DPSCs, trying to uncover the mechanism of osteogenic differentiation of DPSCs for future clinical treatment of bone regeneration.

## 2. Materials and Methods

### 2.1. Isolation and Culture of DPSCs

The tooth removal surgeries were performed at the Affiliated Stomatology Hospital of Kunming Medical University, Kunming, Yunnan, China. The pulp tissues were harvested from one healthy patient, aged 20 years old, with impact third molars, and the removed teeth were free of periodontal or endodontic problems. The removed teeth were stored in precooled PBS immediately, and the next procedures were taken within 4 hours. Under the aseptic condition, the tooth was split and the pulp tissue was removed. Briefly, the pulp tissue was digested for 1 h at 37°C in a solution containing 3 mg/mL collagenase type I and 4 mg/mL dispase. After the filtration through 70 mm cell strainers (Falcon; BD Labware), the cells were cultured at 37°C under 5% CO_2_ in the Dulbecco's modified Eagle's medium (DMEM; Gibco) containing 20% mesenchymal cell growth supplement (Lonza, Inc.) and antibiotics (100 U/mL penicillin, 100 mg/mL streptomycin, and 0.25 mg/mL amphotericin B; Gibco). After 3 days of culture, floating cells were removed and the culture medium was replaced with fresh medium [17].

### 2.2. Identification of DPSCs

The 2^nd^ generation of DPSCs was cultured in a laser confocal dish with 5 × 10^4^ cells. When cells converged to 50%, the culture was terminated and fixed by 4% paraformaldehyde for 15 minutes. PBS with 0.5% Triton was used for 15 minutes, and 5% BSA was used to block for 1 hour. Then, the rabbit antivimentin and mouse anticytokeratin (abcam, US) were added and incubated overnight at 4°C, and PBS washed three times. The FITC-labeled sheep antimouse and cy3-labeled sheep anti-rabbit antibody were added and incubated at the room temperature for 1 hour, then PBS washed three times. Cell nuclei were stained with DAPI, and laser confocal microscopy was used for observation and photographing.

The 2^nd^ generation DPSCs were taken, digested by trypsin, washed by PBS three times, suspended, and counted. The cells were divided into several flow tubes according to the requirement of 1 × 10^5^ cells for each antibody. The cells were incubated with antibodies CD34, CD44, CD45, CD90, and STRO-1 at the room temperature for 45 minutes. Cell surface markers were identified by the flow cytometry after PBS washing and suspension.

### 2.3. Isolation of Osteogenic-Induced DPSC-Derived Exosomes (OI-DPSC-Ex)

Exosomes secreted by DPSCs during the starvation of 48 h without FBS, which were marked as EX0. Other groups of DPSCs were cultured in the osteogenic induction medium (100 nm dexamethasone, 10 mM b-glycerophosphate, and 200 mM ascorbate phosphate in DMEM+10%) with 15% exosome-free FBS (VivaCell, China); exosomes secreted by these osteogenic-induced DPSCs at days 5 and 7 were extracted and marked as EX5 and EX7. The exosomes of EX0, EX5, and EX7 were sent for high-throughput transcriptome sequencing (Guangzhou RiboBio Co., Lt. China) and applied to coculture with homotypic DPSCs. The isolation of exosomes was done followed by the ultracentrifugation method [18]. Briefly, the collected culture medium was centrifugated at 3000g for 20 minutes, and the supernatant was collected. Then, centrifugation at 16500g for 20 minutes, the supernatant was collected again. The supernatant was filtered with a 0.2-micron filter to collect the filtrate. After that, the filtrate was centrifuged at 100,000g for 70 minutes (CP 100WX, Hitachi, Japan), discarded the supernatant, and resuspended the precipitate with PBS. Finally, the precipitation was resuspended with 200 *μ*L PBS.

### 2.4. BCA Test for Exosomes

For quantification and normalization of exosome-containing PBS solutions, the protein in the solutions was quantified by protein BCA Assay Kit (Solarbio, China). The test was performed following the instruction. The finial exosome-containing PBS solutions for next experiments had the protein contained 1 mg/mL.

### 2.5. Transmission Electron Microscopy (TEM)

The EX0, EX5, and EX7 were loaded onto the formvar carbon-coated grids (Ted Pella Inc.), fixed in 2% formaldehyde, and washed. The exosomes were postfixed in 2.5% glutaraldehyde, washed, contrasted with 2% uranyl acetate, and air-dried before TEM examination (Jem 2100, Jeol, Japan).

### 2.6. Nanoparticle Tracking Analysis (NTA) and Flow Cytometry Assay for Exosomes

The exosome particle size of EX0, EX5, and EX7 was verified by analysis of ZETASIZER Nanoseries-Nano-ZS (Malvern, UK) according to the operations manual. For the flow cytometry assay, the EX0, EX5, and EX7 were stained with CD63-FITC and CD81-FITC flow cytometry antibodies (BD Biosciences, San Jose, USA). Then, the nonstained EX0, EX5, and EX7 samples as negative control were marked as NC. The flow cytometry assay was performed according to instrument (BD accuri C6 flow cytometer) operation rules.

### 2.7. DPSCs Cocultured with EX0 and EX7

In this study, the exosomes EX0, EX5, and EX7 were collected and sequenced. EX7 showed higher expression of circLPAR1 than EX5. Therefore, we chose EX7 for subsequent experiments instead of EX5. The 3^rd^ generation of DPSCs was inoculated into 6-well plates with 2 × 10^5^ cells per well density after the cell counting. All wells were randomly divided into EX0-treated group, EX7-treated group, the osteogenic induction medium group as a positive control (PC) group, and normal-cultured group as a negative control (NC) group. For EX0- and EX7-treated groups, DPSCs were cocultured with exosome contained medium (20 *μ*L EX0 or EX7 in 1 mL DMEM+10% exosome-free FBS) without the osteogenic induction medium for 14 days. For PC group, osteogenic differentiation of DPSCs was induced by the osteogenesis induction medium (abovementioned) with 15% exosome-free FBS for 14 days. The NC group was normal-cultured DPSCs (DMEM+15% exosome-free FBS). All the culture medium was replaced, and morphological changes were observed under the microscope every 3 days.

### 2.8. Exosome Phagocytosis

Phagocytosis of exosomes was detected by the following method: DPSCs were inoculated into 12-well plates with 3 × 10^4^ cells per well (15% exosome-free FBS+DMEM were used for culture). The 20 *μ*L exosome solution with protein contained 1 *μ*g/*μ*L was mixed to 4 *μ*L PKH67 and 200 *μ*L diluent, and incubated at the room temperature for 5min. Next, 200 *μ*L exosome-free FBS was added to terminate the reaction; then, the exosomes were extracted. DPSCs were inoculated into 12-well plates with 10% exosome-free FBS+DMEM and divided into the control group and experimental group. The control group was added with the exosomes no marking of PKH67 and 15% exosome-free FBS+DMEM. The experimental group was added with the exosomes marked by PKH67 and 15% exosome-free FBS, incubating at 37°C and 5% CO_2_ for 24 hours. Then, the original culture medium was removed, washed twice with PBS, fixed with 4% paraformaldehyde at the room temperature for 30 min, washed twice with PBS, and stained with DAPI. The phenomenon of phagocytosis of exosomes was observed by a fluorescence microscope (TE2000U, Nikon, Japan).

### 2.9. Alkaline Phosphatase (ALP) Staining and Alizarin Red Staining (ARS)

ALP staining was performed for all groups on day 0, day 7, and 14 incubation. The ALP dye solution was prepared according to the instructions of alkaline phosphatase kit (Colorimetric, abcam, US). The positive results of ALP staining were blue. Secondly, ARS was performed on day 0, day 7, day 14, and 21 for all groups. Briefly, 0.1% alizarin red staining solution was incubated at 37°C for 30 minutes; then, the cells were rinsed by distilled water gently. After drying, mineralized nodules were observed under the microscope and photographed.

### 2.10. Western Blotting

WB assay was performed in the miR-31 knockdown group (si-miR-31), circ lysophosphatidic acid receptor 1- (circLPAR1-) overexpressed group (circLPAR1), EX0-, and EX7-treated groups. Equal amounts of total sample protein were separated by SDS-PAGE and blotted onto PVDF membranes (Millipore). Then, the membranes were immunoblotted with the primary antibody at 4°C for 16 h. The primary antibodies of special AT-rich sequence-binding protein 2 (SATB2) (abcam, ab92446), RUNX2 (abcam, ab76956), col-1 (abcam, ab138492), and OCN (abcam, ab13420) were used. Next, the membrane was washed and incubated with an HRP-conjugated secondary antibody (abcam) at RT for 2 h. All protein signals were analyzed with the ECL Kit (Pierce, US).

### 2.11. Reverse Transcription PCR

The expression level of circLPAR1, hsa-miR-31, and osteogenesis-related genes was detected by reverse transcription PCR (RT-PCR). The Trizol kit (Thermo, US) was used to extract total RNA, and reverse transcriptase (Takara, US) was used to synthesize DNA template. The primers were synthesized from Sangon Biotech (Shanghai, China), and the sequence of PCR primers was as follows: RUNX2 (F: TGGTTACTGTCATGGCGGGTA; R: TCTCAGATCGTTGAACCTTGCTA), col-1 (F: GAGGGCCAAGACGAAGACATC; R: CAGATCACGTCATCGCACAAC), OCN (F: AGCCCATTAGTGCTTGTAAAGG; R: CCCTCCTGCTTGGACACAAAG), CircLPAR1 (F: GGAATCGGGATACCATGATGAGTCT; R: CAGGTACTCAGATAGGTGGATGGGG), and GAPDH (F: CAGGGCTGCTTTTAACTCTGG; R: TGGGTGGAATCATATTGGAACA). The fluorescence quantitative PCR experiment was carried out with MX300p quantitative PCR instrument, and the CT values of each template were detected. Relative quantification was carried out by detecting CT values, and the contents of RUNX2, col-1, OCN, circLPAR1 (hsa_circ_0003611), and GAPDH genes in samples were detected. MiR-31 was reverse transcribed into cDNA using TaqMan® microRNA Reverse Transcription Kit and detected by The TaqMan Universal PCR Master Mix II (Applied Biosystems). U6 and GAPDH were used as endogenous control.

### 2.12. Vector Construction and Cell Transfection

The genomic region of circLPAR1 (hsa_circ_0003611) was synthesized by Sangon Biotech (Shanghai, China) and subcloned into a pcDNA3.1 vector. hsa-miR-31 inhibitor was supplied by Sangon Biotech. Transfection of circPLAR1-PCDNA plasmid and hsa-miR-31 inhibitor was used Lipofectamine 3000 (Thermo) reagent according to the instructions and cultured for 14 days. The hsa-miR-31 inhibitors were repeatedly transfected every 3 days.

### 2.13. Luciferase Reporter Assay

For luciferase assays, wild-type circLPAR1 (hsa_circ_0003611) and mutant type of the binding site genomic region fragments were synthesized by Sangon Biotech (Shanghai, China) and inserted into PmirGLO vector. hsa-miR-31 mimics were supplied by Sangon Biotech. The activity of Firefly luciferase and Renilla luciferase was detected with Dual-Luciferase® Reporter Assay Systems (Promega). Every analysis was performed three times.

### 2.14. Statistical Analysis

All experiments were performed at least three times. The data were represented as the mean ± standard deviation (mean ± SD). Data were analyzed using the Student's two-tailed *t*-test to compare the means of two groups or a one-way ANOVA for comparison of the means of more than two groups using SPSS 17.0 (IBM, Chicago, IL, USA). *P* < 0.05 was considered statistically significant.

### 2.15. Ethics Statement

This study was approved by the Medical Ethics Committee of Kunming Medical University. The patients who donated pulp tissues during the tooth removal surgeries signed the informed consent.

## 3. Results

### 3.1. Isolation and Characterization of DPSCs

**Figure 1:**
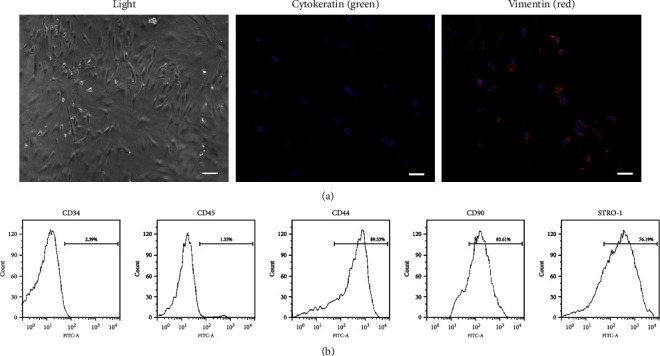
Characterization of DPSCs by (a) immunofluorescence staining and (b) flow cytometry assay. (a) DPSCs were stained with vimentin (red) and cytokeratin (green). Nucleus was stained by DAPI (blue). (b) DPSCs were stained with endothelial-specific markers (CD34 and CD45) and mesenchymal-specific markers (CD44, CD90, and STRO-1).

Immunofluorescence staining of DPSCs showed positive expression of mesenchymal marker vimentin (red) and negative expression of cytokeratin (green) (Figure 1(a)). Flow cytometry assay showed that mesenchymal-specific markers CD44, CD90, and STRO-1 were expressed positively, and the expression rate of cell surface antigen was close to 85%. Hematopoietic and endothelial specific antigens CD34 and CD45 were expressed negatively (Figure 1(b)).

### 3.2. Identification of Exosomes

**Figure 2:**
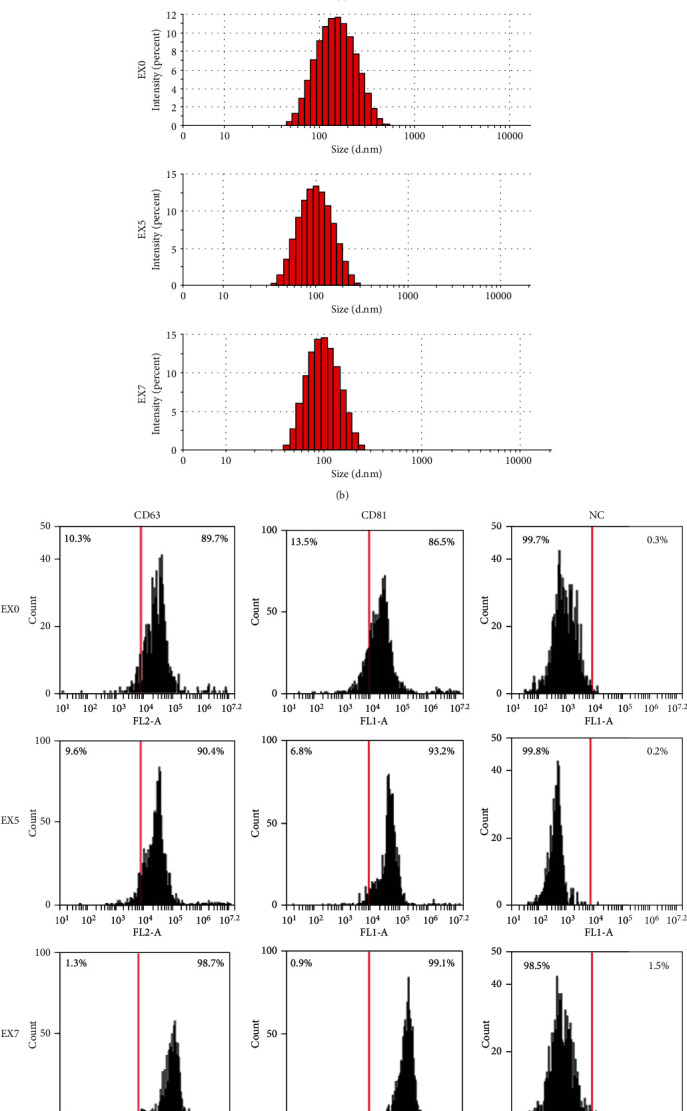
Identification of exosomes by (a) transmission electron microscopy, (b) nanoparticle tracking analysis, and (c) flow cytometry assay. (a) Electron micrographs showed that all exosomes detected were circular structures with a size range of 20-120 nm. (b) The average particle size and main peak of particle size were within the range of exosome particle size. (c) The exosome-specific marker (CD63 and CD81) in EX0, EX5, and EX7 was detected by flow cytometry. The NC group was exosomes with no stain.

Exosomes were isolated from starvation of DPSCs' culture medium marked as EX0, from DPSCs' osteogenic-induced culture medium at days 5 and 7 marked as EX5 and EX7. All groups of exosomes showed circular structures with a size range of 20-120 nm under TEM scanning (Figure 2(a)). Nanoparticle tracking analysis revealed that the average particle size and main peak of particle size were within the range of exosome particle size (Figure 2(b)). The detected particle distribution coefficient (PDI) was between 0.08 and 0.7, which proved the moderate dispersion of the system and high confidence of the results. The expression of CD63 and CD81 in EX0, EX5, and EX7 was detected by flow cytometry instrument. Compared with the NC group, both two tested markers had highly expressed signals (Figure 2(c)).

### 3.3. Osteogenic-Induced DPSC-Derived Exosomes (OI-DPSC-Ex) Induced Osteogenic Differentiation of Recipient Homotypic DPSCs

**Figure 3:**
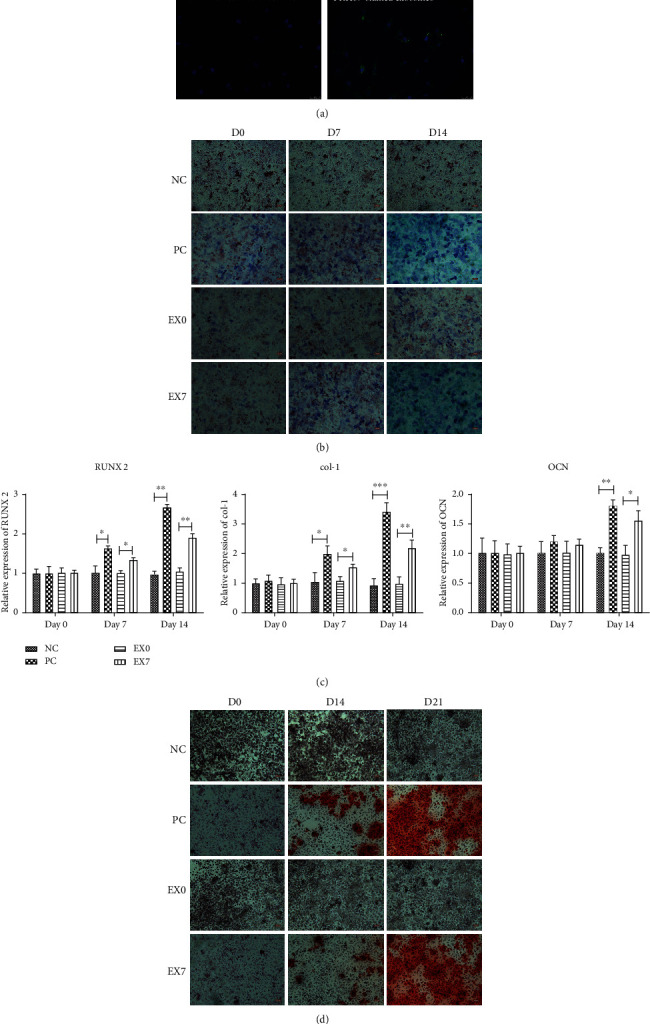
Evaluation of osteogenic differentiation of DPSCs after exosome treatment. (a) PKH67-stained exosomes were phagocytosed by DPSCs (green); no PKH67-stained signal was found in the negative control; nucleus was stained by DAPI (blue). (b) ALP assay of osteogenesis induction medium groups (PC) and exosome-treated group (EX0 and EX7) cultured at days 0 (D0), 7 (D7), and 14 (D14). (c) The expression levels of RUNX2, col-1, and OCN before and after exosome induction were detected by qRT-PCR. (d) Alizarin red staining of PC group and exosome-treated groups (EX0 and EX7) cultured at days 0 (D0), 7 (D7), 14 (D14), and 21 (D21). NC was the negative control. ^∗^*P* < 0.05, ^∗∗^*P* < 0.01, and ^∗∗∗^*P* < 0.001 indicated significant differences.

The fluorescence microscope demonstrated that the exosomes were phagocytized by DPSCs (Figure 3(a)). Subsequently, we tested the osteogenic effects of EX0 and EX7 on homotypic DPSCs. ALP activity in PC groups at days 7 (D7) and 14 (D14) was significantly higher than that at the initial time point day 0 (D0). The exosome induction group EX7 also showed similar staining results (Figure 3(b)). ARS confirmed that the EX7 group produced the calcium deposit by recipient DPSCs (Figure 3(d)). The highly expressed level of osteogenic induction genes RUNX2, col-1, and OCN further confirmed that EX7 promoted osteogenic differentiation of recipient homotypic DPSCs (Figure 3(c)). In contrast to EX7 group, EX0 group showed similar results in ALP staining, ARS, and level of osteogenic induction gene expression, which was considered had no osteogenic effect on DPSCs.

### 3.4. CircLPAR1 Was Obviously Upregulated in Osteogenic-Induced DPSC-Derived Exosomes

**Figure 4:**
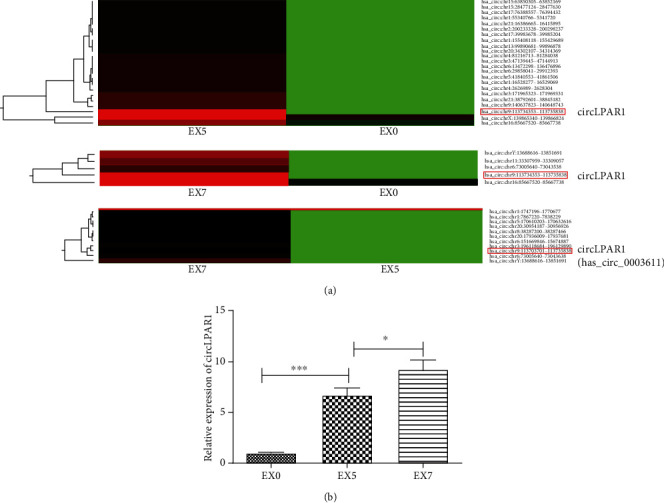
Exosomal circRNA profiles during osteogenic differentiation of DPSCs. (a) Altered circRNA profiles of exosomes during osteogenic differentiation time intervals (EX0, EX5, and EX7). Red color represented an expression level above the mean, and green color represented expression lower than the mean. (b) The expression level of exosomal circLPAR1 during osteogenic differentiation time intervals (EX0, EX5, and EX7) was detected by qRT-PCR. ^∗^*P* < 0.05, ^∗∗^*P* < 0.01, and ^∗∗∗^*P* < 0.001 indicated significant differences.

To investigate the mechanism of OI-DPSC-Ex on promoting osteogenic differentiation of DPSCs, we performed high-throughput sequencing of circRNA in EX0, EX5, and EX7. Through bioinformatics analysis of the sequencing result, we found that there were 11 circRNAs raised steadily from EX5 to EX7 (Figure 4(a)). By fluorescence quantitative PCR detection, we confirmed that LPAR1 (hsa_circ_0003611) expression level in exosomes was increased gradually with the extension of induction time (Figure 4(b)). Therefore, we listed LPAR1 as a research object, hoping to explore the physiological role of circLPARP1 in exosomes.

### 3.5. hsa-miR-31 Was the Target of circLPAR1

**Figure 5:**
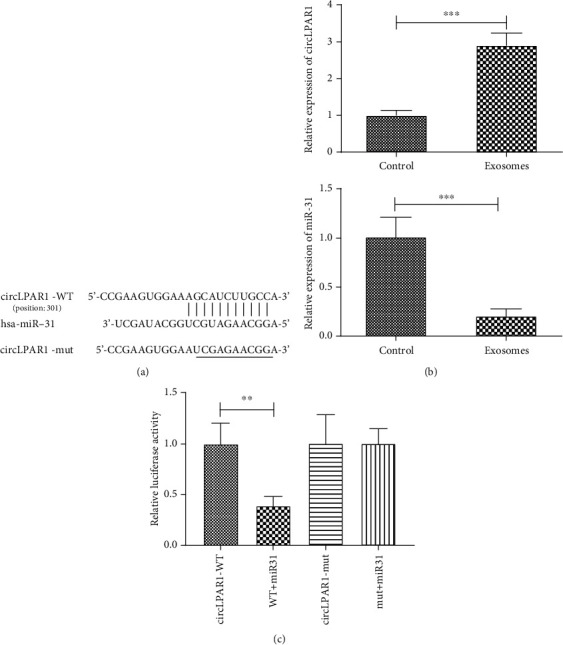
CircLPAR1 bound to hsa-miR-31 and inhibited its activity. (a) The sequences of hsa-miR-31 and predicted binding sites in the circLPAR1. (b) The expression levels of hsa-miR-31 and circLPAR1 in EX0- and EX7-induced DPSCs. (c) Relative luciferase activity of circLPAR1-WT, WT+miR-31 mimics, circLPAR1 mutant, and mutant+miR-31 mimics was detected. ^∗^*P* < 0.05, ^∗∗^*P* < 0.01, and ^∗∗∗^*P* < 0.001 indicated significant differences.

It was predicted by online tools (https://circinteractome.nia.nih.gov/) that circLPAR1 (hsa_circ_0003611) bound to hsa-miR-31, a miRNA that showed significant inhibitory effect on osteogenic differentiation [19, 20] (Figure 5(a)). In order to confirm the accuracy of this prediction, verified experiments were conducted. First, we detected the expression levels of hsa-miR-31 and circLPAR1genes in EX7- and EX0-treated DPSCs. The results showed that circLPAR1 expression level was upregulated in EX7-treated DPSCs, while hsa-miR-31 was decreased significantly (Figure 5(b)). There was a negative correlation between the two RNAs. Furthermore, we verified whether circPLAR1 was a direct hsa-miR-31 target using luciferase reporter assays. DPSCs cotransfected with the hsa-miR-31 mimics, and circPLAR1 plasmid suppressed the activity of a luciferase reporter, but did not affect the mutant circLPAR1 group. This result demonstrated that circLPAR1 would be the target of hsa-miR-31 (Figure 5(c)).

### 3.6. Exosomal circLPAR1 Induced Osteogenic Differentiation via Downregulation of hsa-miR-31

**Figure 6:**
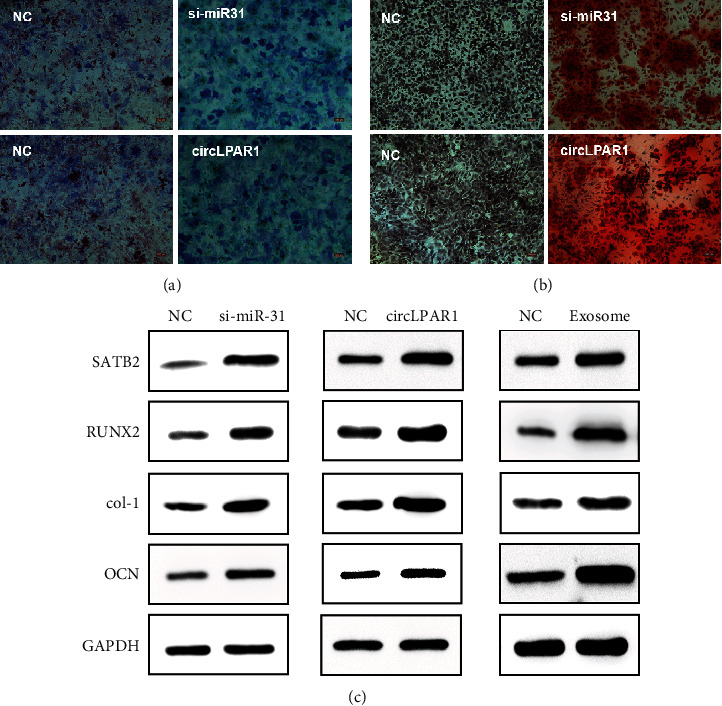
CircLPAR1 in exosomes induced osteogenic differentiation of recipient homotypic DPSC through downregulation of miR-31. (a) ALP assay of miR-31-inhibited DPSCs and circLPAR1-overexpressed DPSCs cultured for 14 days. (b) Alizarin red staining of miR-31-inhibited groups and circLPAR1-overexpressed group cultured for 21 days. (c) The expression level of important osteogenic differentiation-related genes (SATB2, RUNX2, col-1, and OCN) in miR-31-inhibited group, circLPAR1 overexpressed, and EX7 treated for 14 days. DPSCs were detected by Western blot assay.

Based on above results, we hypothesized that circLPAR1 induced osteogenic differentiation of DPSCs through competitively binding to hsa-miR-31. To verify this hypothesis, we transfected hsa-miR-31 inhibitor and circLPAR1 overexpression vector into DPSCs. ALP activity detection and alizarin red staining were performed on the 14^th^ day and 21^st^ day after the transfection. We found that both downregulation of hsa-miR-31 and upregulation of circLPAR1 promoted osteogenic differentiation of DPSCs by the ALP assay and ARS (Figures 6(a) and 6(b)). The WB assay was used to detect the expression of SATB2, RUNX2, col-1, and OCN in si-miR-31, circLPAR1 overexpression, and EX7-treated groups for 14 days. Compared with the NC group, the expression of osteogenic differentiation-related proteins in the three experimental groups was all elevated (Figure 6(c)).

## 4. Discussion

Decoding the mystery of osteogenic differentiation would be the cardinal step for archiving predictable bone regeneration outcomes. However, due to various trigger factors, the complex of signaling pathways, etc., osteogenic differentiation remains many unanswered questions. In recent decades, DPSC has been chosen as a promising cell source for regenerative medicine [3]. However, the clinical application of DPSC is still far from ideal [21]. It might be due to a major reason of unclear mechanism of osteogenic differentiation of DPSCs.

The discovery of exosome has opened a new direction of cell research, particularly the cell-cell communications [22]. Moreover, the exosome played important roles in cellular differentiation [23]. On the one hand, the biomolecular messages inside of exosomes altered synchronously to the stages of osteogenic differentiation. Xu et al. reported the alteration of exosomal miRNA expression during the osteogenic differentiation and the different expression correlated to the degrees of osteogenic differentiation [7]. On the other hand, the altered biomolecular messages loaded in exosomes had specific biological effects closely related to its ongoing osteogenic differentiation. Wang et al. not only showed the change of exosomal miRNA expression during the osteogenic differentiation, but they also demonstrated the exosomes from various stages of osteogenic differentiation that had different osteogenic effects on the homotypic recipient cells [6]. In addition, it was found that the exosomes from osteogenic-induced stem cells from human exfoliated deciduous teeth contained mRNA and proteins of Wnt3a and BMP2, which showed osteogenic effects on periodontal ligament stem cells (PDLSCs) [24]. However, those are the researches of exosomal miRNA, mRNA, and proteins related to osteogenic differentiation.

Recently, circRNA has showed the impact on osteogenic differentiation. Lloret-Llinares et al. found different expression of certain circRNAs during osteogenic differentiation of MC3T3-E1 cells by RNA sequencing (RNA-seq), and they demonstrated a circ19142/circ5846-targeted miRNA–mRNA axis [25]. A thorough analysis of circRNA expression profiles during osteogenic differentiation of PDLSCs revealed that more than one hundred circRNAs changed expression significantly and showed a stage-specific change of circRNA expression [26]. 43 circRNAs were found to change expression during osteogenic differentiation of mouse adipose-derived stromal cells, of which two circRNAs (mmu_circRNA_013422 and mmu_circRNA_22566) were upregulated and showed the miRNA-sponge effect to miR-338-3p [27]. Zhang et al. had verified a link between circIGSF11 and miR-199b-5p, the downregulation of circIGSF11 led to enhancement of osteogenesis by the upregulation of miR-199b-5 expression [14]. Those studies were of importance to show the role of circRNAs in osteogenesis. However, up to date, very few studies considered the exosomal circRNA, which is the special cargo of exosome having effects on the recipient cells for osteogenesis. Hence, our study firstly reported the altered expression of exosomal circRNA of DPSCs undergoing osteogenic differentiation and identified the circLPAR1 that played a promotive role on osteogenic differentiation of DPSCs.

We thoroughly examined all exosomal circRNAs by RNA-seq at days 5 (EX5) and 7 (EX7) after osteogenic induction and day 0 (EX0, the exosomes from starvation of DPSCs without osteogenic induction). Among upregulated exosomal circRNAs, circLPAR1 was continuously upregulated along with induction time as the results of comparing EX5, EX7 to EX0, respectively. However, EX7 had a higher expression of circLPAR1 than EX5. Therefore, the EX7 was selected for testing its osteogenic effects. The results of EX7 showed similar effects as the positive control group, whereas the EX0 group showed the null effect on osteogenesis. EX7 contained the high level of circLPAR1 expression would be the key point of promotion of osteogenesis of DPSCs. CircLPAR1 is a kind of G-protein coupled receptor commonly expressed in normal human tissues [28], and it has been studied in cancer field recently [29–31], but its effect on osteogenesis remains mostly unknown. The results from bioinformatic prediction and luciferase reporter assay showed that circLPAR1 had a strong binding capacity with hsa-miR-31 which was a proved miRNA inhibitor of osteogenic differentiation [32–34]. Moreover, hsa-miR-31 inhibited the osteogenic differentiation of mesenchymal stem cells by targeting SATB2 [20, 34, 35], a protein that showed significant role in bone biology and positively linked to the level of expression of RUNX2, OPN, OSX, OCN, etc. [12, 19, 20, 35–37]. In this study, the results confirmed that the expression level of SATB2 and other osteogenic differentiation-related genes was upregulated in the exosome (EX7) treated, si-miR-31 transinfected, and circLPAR1-overexpressed groups.

**Figure 7:**
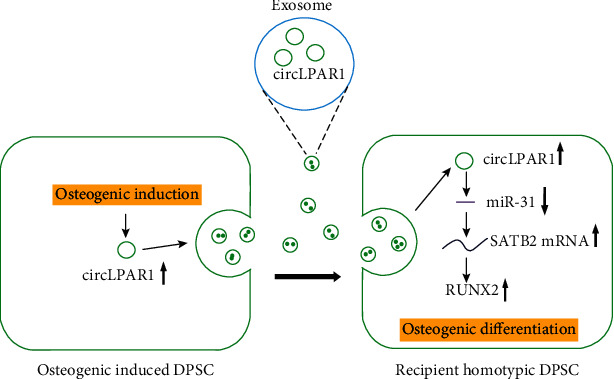
Overview hypothesis of the present study: exosomes derived from osteogenic-induced DPSCs rich in circLPAR1; then, the exosomes were phagocytosed by the recipient homotypic DPSCs and lift up the expression level of circLPAR1 in the recipient cells. Afterwards, circLPAR1 could competently bind to miR-31, thereby eliminating the inhibitory effect of miR-31 on osteogenic differentiation, as a consequence of promoting osteogenic differentiation of the recipient homotypic DPSCs.

Our study identified that circLPAR1 was highly expressed in the exosomes derived from osteogenic-induced DPSCs. Then, the large number of circLPAR1 entered the recipient homotypic DPSCs, then bound to hsa-miR-31 which was the miRNA targeted to gene SATB2. Therefore, circLPAR1 eliminated negative effect of hsa-miR-31 on osteogenic differentiation of DPSCs. Subsequently, the expression level of SATB2 was increased and led to the upregulation of its downstream genes which related to osteogenic differentiation such as RUNX2. The increased RUNX2 promoted the occurrence and development of osteogenic differentiation (Figure 7).

The highlight of this study was to investigate the effects of OI-DPSC-Ex on homotypic DPSCs, which showed one possible role of the exosomes played during osteogenic differentiation. We supposedly considered the effect of the exosomes derived from induced cells further inducing the homotypic cells as a “re-enhanced” loop or a chain reaction or a “turbocharger-effect”; these exosomes further amplified the induction effect on themselves and surrounding cells as a consequence of a successful completion of differentiation.

## 5. Conclusion

This study demonstrated the increasing circLPAR1 expression in the exosomes derived from DPSCs during osteogenic differentiation. These exosomes had the osteogenic effect on the recipient homotypic DPSCs via exosomal circLPAR1 that upregulated SATB2 expression by competitively binding to hsa-miR-31. Our findings uncovered exosomal circRNA expression profile during osteogenic differentiation of DPSCs and revealed a new mode of understanding of the role of exosomes played in osteogenic differentiation, providing a novel way of utilization of exosomes for the treatment of bone deficiencies.

## Data Availability

The data are available upon reasonable request.

## Supplementary Materials

Supplementary Materials Original sequencing data and analysis of exosomes derived from DPSCs during osteogenic differentiation.
