# Supplementary material for: Exosomal circLPAR1 Promoted Osteogenic Differentiation of Homotypic Dental Pulp Stem Cells by Competitively Binding to hsa-miR-31
Source: Biomed Res Int. 2020 Sep 28;2020:6319395. doi: 10.1155/2020/6319395 (PMC7539105; doi:10.1155/2020/6319395)
Supplement: Supplementary Materials — Original sequencing data and analysis of exosomes derived from DPSCs during osteogenic differentiation. [file 6319395.f1.zip › Original Data and Analysis of DPSC' Exosomes Sequencing/(D5-1) VS (D7-1)/3. D5-1--D7-1.circRNA.heatmap.pdf]

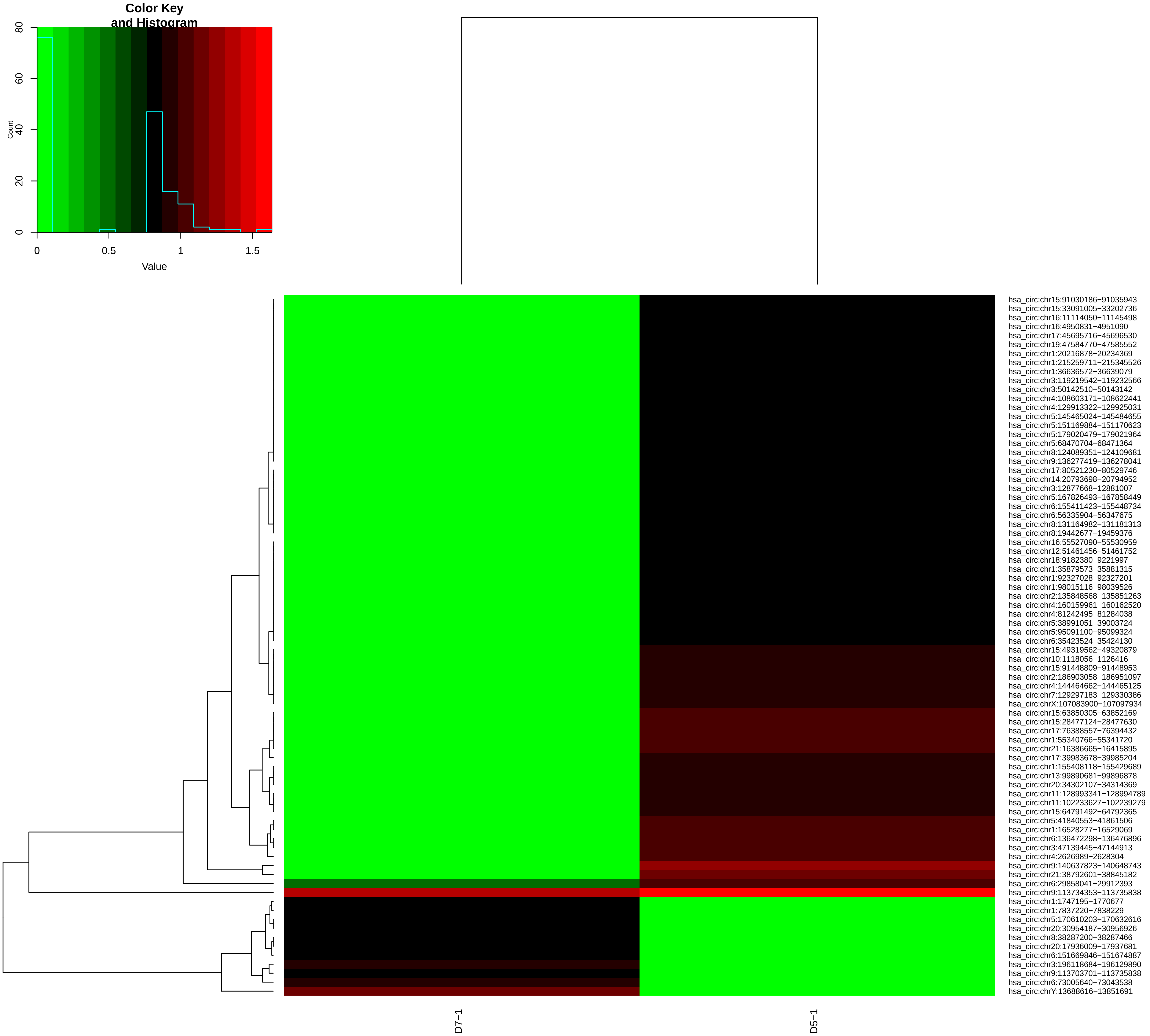
Color Key
and Histogram
Count
0
20
40
60
80
0
0.5
1
1.5
Value
hsa_circ:chr15:91030186−91035943
hsa_circ:chr15:33091005−33202736
hsa_circ:chr16:11114050−11145498
hsa_circ:chr16:4950831−4951090
hsa_circ:chr17:45695716−45696530
hsa_circ:chr19:47584770−47585552
hsa_circ:chr1:20216878−20234369
hsa_circ:chr1:215259711−215345526
hsa_circ:chr1:36636572−36639079
hsa_circ:chr3:119219542−119232566
hsa_circ:chr3:50142510−50143142
hsa_circ:chr4:108603171−108622441
hsa_circ:chr4:129913322−129925031
hsa_circ:chr5:145465024−145484655
hsa_circ:chr5:151169884−151170623
hsa_circ:chr5:179020479−179021964
hsa_circ:chr5:68470704−68471364
hsa_circ:chr8:124089351−124109681
hsa_circ:chr9:136277419−136278041
hsa_circ:chr17:80521230−80529746
hsa_circ:chr14:20793698−20794952
hsa_circ:chr3:12877668−12881007
hsa_circ:chr5:167826493−167858449
hsa_circ:chr6:155411423−155448734
hsa_circ:chr6:56335904−56347675
hsa_circ:chr8:131164982−131181313
hsa_circ:chr8:19442677−19459376
hsa_circ:chr16:55527090−55530959
hsa_circ:chr12:51461456−51461752
hsa_circ:chr18:9182380−9221997
hsa_circ:chr1:35879573−35881315
hsa_circ:chr1:92327028−92327201
hsa_circ:chr1:98015116−98039526
hsa_circ:chr2:135848568−135851263
hsa_circ:chr4:160159961−160162520
hsa_circ:chr4:81242495−81284038
hsa_circ:chr5:38991051−39003724
hsa_circ:chr5:95091100−95099324
hsa_circ:chr6:35423524−35424130
hsa_circ:chr15:49319562−49320879
hsa_circ:chr10:1118056−1126416
hsa_circ:chr15:91448809−91448953
hsa_circ:chr2:186903058−186951097
hsa_circ:chr4:144464662−144465125
hsa_circ:chr7:129297183−129330386
hsa_circ:chrX:107083900−107097934
hsa_circ:chr15:63850305−63852169
hsa_circ:chr15:28477124−28477630
hsa_circ:chr17:76388557−76394432
hsa_circ:chr1:55340766−55341720
hsa_circ:chr21:16386665−16415895
hsa_circ:chr17:39983678−39985204
hsa_circ:chr1:155408118−155429689
hsa_circ:chr13:99890681−99896878
hsa_circ:chr20:34302107−34314369
hsa_circ:chr11:128993341−128994789
hsa_circ:chr11:102233627−102239279
hsa_circ:chr15:64791492−64792365
hsa_circ:chr5:41840553−41861506
hsa_circ:chr1:16528277−16529069
hsa_circ:chr6:136472298−136476896
hsa_circ:chr3:47139445−47144913
hsa_circ:chr4:2626989−2628304
hsa_circ:chr9:140637823−140648743
hsa_circ:chr21:38792601−38845182
hsa_circ:chr6:29858041−29912393
hsa_circ:chr9:113734353−113735838
hsa_circ:chr1:1747195−1770677
hsa_circ:chr1:7837220−7838229
hsa_circ:chr5:170610203−170632616
hsa_circ:chr20:30954187−30956926
hsa_circ:chr8:38287200−38287466
hsa_circ:chr20:17936009−17937681
hsa_circ:chr6:151669846−151674887
hsa_circ:chr3:196118684−196129890
hsa_circ:chr9:113703701−113735838
hsa_circ:chr6:73005640−73043538
hsa_circ:chrY:13688616−13851691
D7−1
D5−1
