# Supplementary material for: Exosomal circLPAR1 Promoted Osteogenic Differentiation of Homotypic Dental Pulp Stem Cells by Competitively Binding to hsa-miR-31
Source: Biomed Res Int. 2020 Sep 28;2020:6319395. doi: 10.1155/2020/6319395 (PMC7539105; doi:10.1155/2020/6319395)
Supplement: Supplementary Materials — Original sequencing data and analysis of exosomes derived from DPSCs during osteogenic differentiation. [file 6319395.f1.zip › Original Data and Analysis of DPSC' Exosomes Sequencing/(DC-1) VS (D5-1)/3. DC-1 VS D5-1.circRNA.heatmap.pdf]

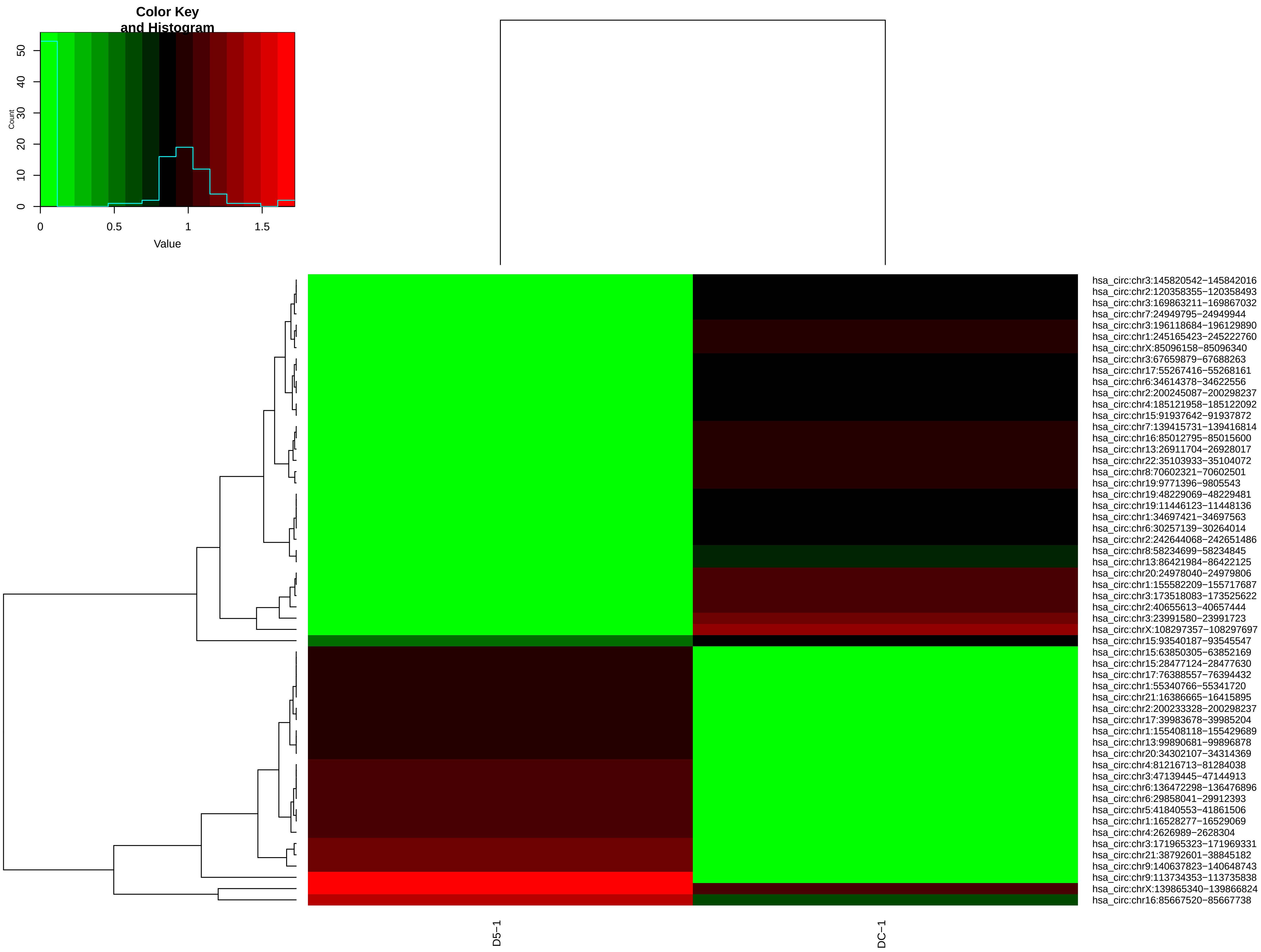
Color Key
and Histogram
Count
0
10
20
30
40
50
0
0.5
1
1.5
Value
hsa_circ:chr3:145820542−145842016
hsa_circ:chr2:120358355−120358493
hsa_circ:chr3:169863211−169867032
hsa_circ:chr7:24949795−24949944
hsa_circ:chr3:196118684−196129890
hsa_circ:chr1:245165423−245222760
hsa_circ:chrX:85096158−85096340
hsa_circ:chr3:67659879−67688263
hsa_circ:chr17:55267416−55268161
hsa_circ:chr6:34614378−34622556
hsa_circ:chr2:200245087−200298237
hsa_circ:chr4:185121958−185122092
hsa_circ:chr15:91937642−91937872
hsa_circ:chr7:139415731−139416814
hsa_circ:chr16:85012795−85015600
hsa_circ:chr13:26911704−26928017
hsa_circ:chr22:35103933−35104072
hsa_circ:chr8:70602321−70602501
hsa_circ:chr19:9771396−9805543
hsa_circ:chr19:48229069−48229481
hsa_circ:chr19:11446123−11448136
hsa_circ:chr1:34697421−34697563
hsa_circ:chr6:30257139−30264014
hsa_circ:chr2:242644068−242651486
hsa_circ:chr8:58234699−58234845
hsa_circ:chr13:86421984−86422125
hsa_circ:chr20:24978040−24979806
hsa_circ:chr1:155582209−155717687
hsa_circ:chr3:173518083−173525622
hsa_circ:chr2:40655613−40657444
hsa_circ:chr3:23991580−23991723
hsa_circ:chrX:108297357−108297697
hsa_circ:chr15:93540187−93545547
hsa_circ:chr15:63850305−63852169
hsa_circ:chr15:28477124−28477630
hsa_circ:chr17:76388557−76394432
hsa_circ:chr1:55340766−55341720
hsa_circ:chr21:16386665−16415895
hsa_circ:chr2:200233328−200298237
hsa_circ:chr17:39983678−39985204
hsa_circ:chr1:155408118−155429689
hsa_circ:chr13:99890681−99896878
hsa_circ:chr20:34302107−34314369
hsa_circ:chr4:81216713−81284038
hsa_circ:chr3:47139445−47144913
hsa_circ:chr6:136472298−136476896
hsa_circ:chr6:29858041−29912393
hsa_circ:chr5:41840553−41861506
hsa_circ:chr1:16528277−16529069
hsa_circ:chr4:2626989−2628304
hsa_circ:chr3:171965323−171969331
hsa_circ:chr21:38792601−38845182
hsa_circ:chr9:140637823−140648743
hsa_circ:chr9:113734353−113735838
hsa_circ:chrX:139865340−139866824
hsa_circ:chr16:85667520−85667738
D5−1
DC−1
