# Supplementary material for: Exosomal circLPAR1 Promoted Osteogenic Differentiation of Homotypic Dental Pulp Stem Cells by Competitively Binding to hsa-miR-31
Source: Biomed Res Int. 2020 Sep 28;2020:6319395. doi: 10.1155/2020/6319395 (PMC7539105; doi:10.1155/2020/6319395)
Supplement: Supplementary Materials — Original sequencing data and analysis of exosomes derived from DPSCs during osteogenic differentiation. [file 6319395.f1.zip › Original Data and Analysis of DPSC' Exosomes Sequencing/(DC-1) VS (D7-1)/Caption.docx]

Caption

1. The data showed differential expressed circRNAs between DC-1 and D7-1.

2. The data showed all expressed circRNAs of DC-1 and D7-1.

3. The heatmap compared between DC-1 and D7-1.

4. The volcano compared between DC-1 and D7-1.
