# Supplementary material for: Exosomal circLPAR1 Promoted Osteogenic Differentiation of Homotypic Dental Pulp Stem Cells by Competitively Binding to hsa-miR-31
Source: Biomed Res Int. 2020 Sep 28;2020:6319395. doi: 10.1155/2020/6319395 (PMC7539105; doi:10.1155/2020/6319395)
Supplement: Supplementary Materials — Original sequencing data and analysis of exosomes derived from DPSCs during osteogenic differentiation. [file 6319395.f1.zip › Original Data and Analysis of DPSC' Exosomes Sequencing/Footnote of three abbreviations.docx]

Footnote

Osteogenic induced DPSCs at day 0 (DC-1)

Osteogenic induced DPSCs at day 5 (D5-1)

Osteogenic induced DPSCs at day 7 (D7-1)
