# Supplementary figures and images for: Exosomal circLPAR1 Promoted Osteogenic Differentiation of Homotypic Dental Pulp Stem Cells by Competitively Binding to hsa-miR-31
Source: Biomed Res Int. 2020 Sep 28;2020:6319395. doi: 10.1155/2020/6319395 (PMC7539105; doi:10.1155/2020/6319395)

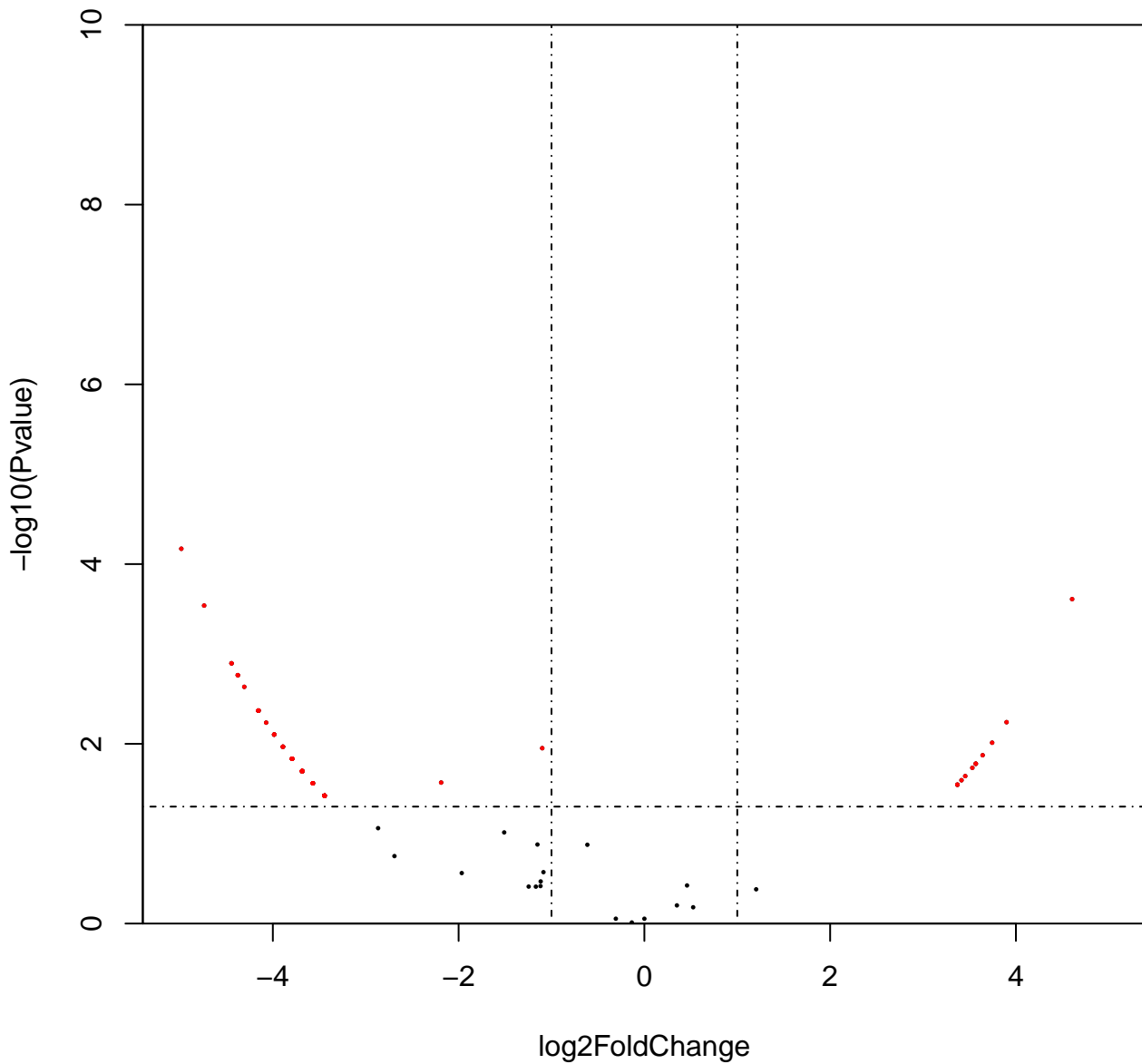

Supplement: Supplementary Materials — Original sequencing data and analysis of exosomes derived from DPSCs during osteogenic differentiation. [file 6319395.f1.zip › Original Data and Analysis of DPSC' Exosomes Sequencing/(D5-1) VS (D7-1)/4. D5-1--D7-1.circRNA.volcano.pdf]

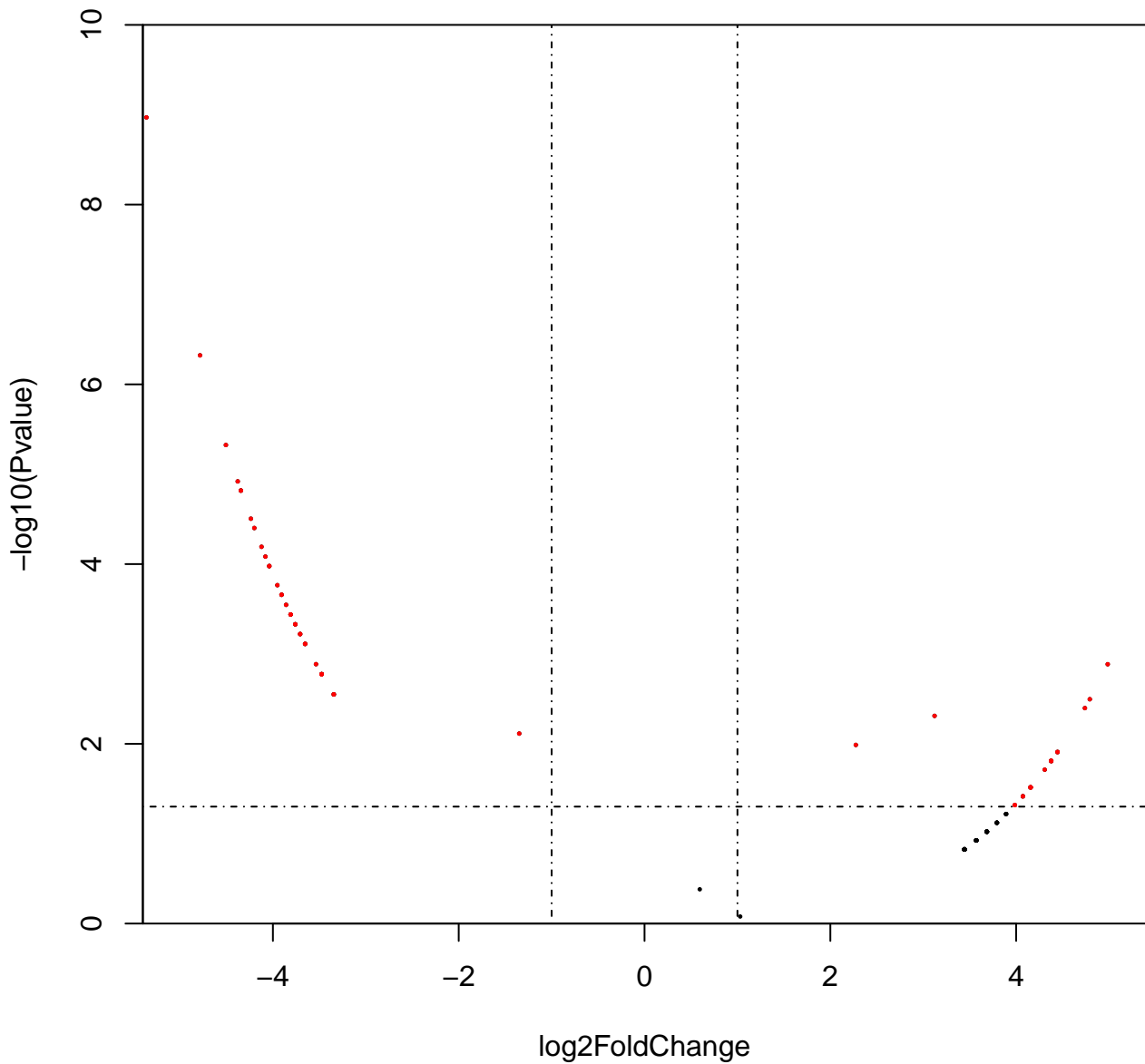

Supplement: Supplementary Materials — Original sequencing data and analysis of exosomes derived from DPSCs during osteogenic differentiation. [file 6319395.f1.zip › Original Data and Analysis of DPSC' Exosomes Sequencing/(DC-1) VS (D5-1)/4. DC-1 VS D5-1.circRNA.volcano.pdf]

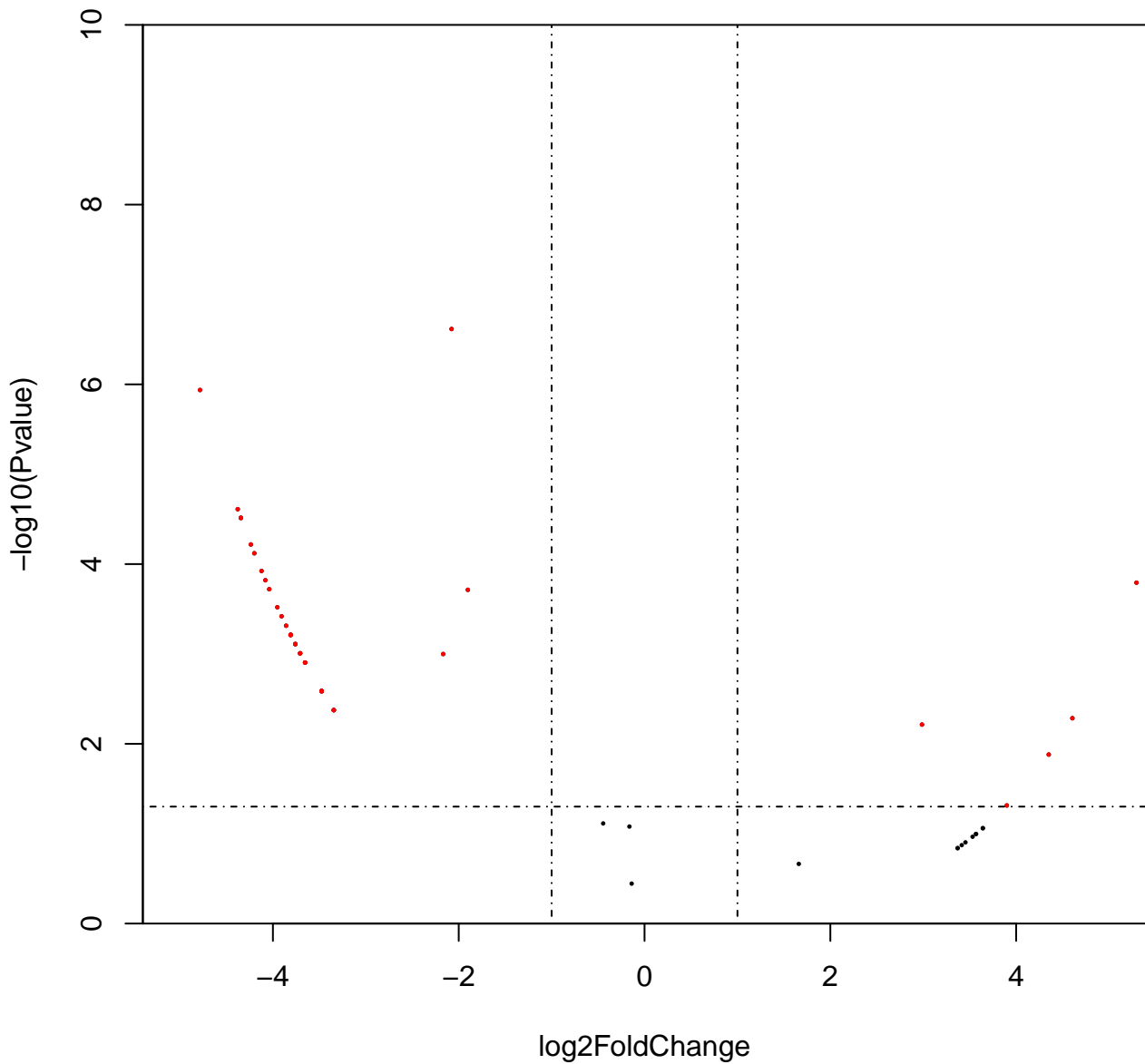

Supplement: Supplementary Materials — Original sequencing data and analysis of exosomes derived from DPSCs during osteogenic differentiation. [file 6319395.f1.zip › Original Data and Analysis of DPSC' Exosomes Sequencing/(DC-1) VS (D7-1)/4. DC-1--D7-1.circRNA.volcano.pdf]
